# Impact of COVID-19 on Healthcare Workers in Brazil between August and November 2020: A Cross-Sectional Survey

**DOI:** 10.3390/ijerph18126511

**Published:** 2021-06-17

**Authors:** Edlaine Faria de Moura Villela, Izadora Rodrigues da Cunha, Joseph Nelson Siewe Fodjo, Michael Obimpeh, Robert Colebunders, Stijn Van Hees

**Affiliations:** 1 Disease Control Coordination, São Paulo State Health Department, São Paulo 01246-000, Brazil; efvillela@saude.sp.gov.br; 2 Institute of Tropical Pathology and Public Health, Federal University of Goiás, Goiânia 74605-050, Brazil; 3 School of Medicine, Health Sciences Unit, Federal University of Jataí, Jataí 75801-615, Brazil; izadoracunha@discente.ufj.edu.br; 4 Global Health Institute, University of Antwerp, 2650 Antwerp, Belgium; josephnelson.siewefodjo@uantwerpen.be (J.N.S.F.); michael.obimpeh@student.uantwerpen.be (M.O.); stijn.vanhees@uantwerpen.be (S.V.H.)

**Keywords:** COVID-19, Brazil, health care workers, anxiety, depression

## Abstract

During the COVID-19 pandemic, healthcare workers (HCW) have been subjected to greater workloads. We conducted a cross-sectional online survey to assess the impact of the COVID-19 pandemic on Brazilian HCW. Data were collected between 11 August and 1 November 2020. Of the 295 respondents, 95 (32.2%) were medical doctors, 82 (27.8%) administrative staff, 53 (18.0%) nurses, 27 (9.2%) laboratory staff, and 38 (12.9%) were other staff. COVID-19-related restructuring at the health facilities was reported by 207 (70.2%) respondents, and 69 (23.4%) had their tasks changed. Preventive measures were well respected when seeing suspected patients. Overall, 167 (56.6%) HCW screened positive for anxiety and 137 (46.4%) for depression; 109 (36.9%) screened positive for both conditions. Of the 217 (73.6%) HCW who had been tested for COVID-19, at least one positive result was reported in 49 (22.6%). Following a positive COVID-19 test, 45/49 (91.8%) stopped working and stayed home. In conclusion, we found a high incidence of COVID-19 infection among Brazilian HCW with high rates of anxiety and depression despite a good self-reported adherence to COVID-19 preventive measures. As such, our study highlights the urgent need for interventions to mitigate the psychosocial risks HCW in Brazil encounter during the COVID-19 pandemic.

## 1. Introduction

With more than 62 million confirmed cases as of 30 November 2020, the coronavirus disease 2019 (COVID-19) pandemic severely impacted all continents around the world [1]. Health systems in many countries were overwhelmed, subjecting healthcare workers (HCW) to increased workloads with inherent pressures. HCW are more likely to be exposed to the virus given their proximity with patients, and many have witnessed unexpected changes in their work routines during the COVID-19 pandemic, including longer working hours and a change in tasks [2,3,4,5]. As such, it was expected that the COVID-19 health crisis would profoundly affect their psychosocial well-being. Indeed, in a recent systematic review and meta-analysis incorporating data from 65 studies with a total of 97,333 HCW, the pooled prevalence of depression and anxiety was 21.7% and 22.1% respectively during the COVID-19 pandemic [6].

With each country having a different course of the pandemic and different restrictive measures imposed by governments, the extent to which HCW might be affected by the COVID-19 varies by country [7,8,9,10,11,12]. Brazil has been severely affected by the COVID-19 pandemic. On 2 November 2020 (around the end of this survey) the country had reported a cumulative 5,631,181 COVID-19 cases, and 162,015 COVID-19 deaths, almost 30% of the number of cases in all the Americas [1]. By that time, the second wave of COVID-19 was yet to start. Driven by the surge of mutant viruses that escape neutralizing antibody responses, the second wave was still ongoing in April 2021 [13]. Hospitals were running out of drugs and oxygen [14]. In the province of Manaus, a resurgence of the pandemic was observed, despite the presence of severe acute respiratory syndrome coronavirus 2 (SARS-CoV-2) antibodies in 76% of the population [15]. In another study performed in Sao Paulo, south-eastern Brazil, in October 2020, the seroprevalence of SARS-CoV-2 was estimated to be 29% [16]. A nation-wide serosurvey showed that the SARS-CoV-2 antibody prevalence was highly heterogeneous by country region [17]. Unfortunately, with the high community transmission rates, a president who is against lockdown measures, and the difficulties in rolling out the COVID-19 vaccination campaign, the end of the COVID-19 crisis in Brazil is not yet in sight [14,15]. 

Given the crucial role of HCW in addressing the COVID-19 pandemic, it is important to understand the impact of this pandemic on HCW’s lives to keep them healthy and effective in the healthcare system. A previous cross-sectional study conducted among a random sample of 536 Brazilian medical doctors, nurses, and dentists during the first six months of 2020 showed that most of them experienced a high level of anxiety and depression [9]. In another study among HCW in Sao Paulo, Brazil, conducted between March and June 2020, evidence of exposure to COVID-19 was documented in 24.1% of the HCW [18]. Both studies suggested an important impact of the COVID-19 pandemic on Brazilian HCW during the first semester of the pandemic. However, as the pandemic progresses, it is important to characterize the medium-to-long-term impact of COVID-19 on the work routine and psychosocial well-being of HCW.

In this study, we assessed the frequency of COVID-19 infection and flu-like symptoms among Brazilian HCW and the impact of the COVID-19 pandemic on their work routine as well as their psychosocial well-being between 11 August and 1 November 2020, in the middle of the first COVID-19 wave, after several months of community transmission.

## 2. Materials and Methods

### 2.1. Study Setting and Design

This was a cross-sectional online questionnaire survey conducted in Brazil between 11 August and 1 November 2020. HCW were invited via social media to anonymously respond to the questionnaire, which was hosted on the secure web platform of the International Citizen Project on Covid-19 (www.icpcovid.com). The questionnaire was developed by the ICPcovid consortium in English, adapted for the Brazilian context, translated in Portuguese, and pilot tested before dissemination. Upon submitting responses, participants were also encouraged to further disseminate the survey link in their networks. The study was conducted in accordance with the Declaration of Helsinki and the protocol was approved by the National Research Ethics Commission, Brazil (CAAE: 30343820.9.0000.0008, n. 4.157.422) and the Antwerp University Hospital (Reference number 18/13/148), Belgium. 

### 2.2. Survey

A 66 item, structured questionnaire was developed to investigate details about the work routine of HCW during the COVID-19 pandemic, the impact of the pandemic on their psychosocial well-being, and information about their own infection status (Table S2). We hypothesized that the COVID-19 pandemic would have had an important impact on both the work routine of Brazilian HCW as well as their psychosocial well-being; this all coinciding with a high rate of COVID-19 infections. Participants were screened for anxiety and depression using the validated hospital anxiety and depression scale (HADS), which has been widely used to assess these conditions during the COVID-19 pandemic [19,20,21,22,23]. The HADS scale consisted of two main sections of seven questions each, one section screening for anxiety and the other for depression. Each answer was given in a 0–3 Likert scale, and participants with a HADS score of eight and above in each section were considered as screening positive for the condition (anxiety or depression). This cut-off value was based on previous studies conducted among HCW during the COVID-19 pandemic [24,25]. 

Using the self-reported flu-like symptoms of the participants, we applied the World Health Organization’s clinical definition for suspected COVID-19 to estimate the disease burden among HCW in Brazil [26]. 

Participants were given the option to leave their email addresses at the end of the questionnaire. Participants who left their email addresses were contacted again in January 2021 with a small questionnaire (Table S3) to assess their willingness to receive the COVID-19 vaccine. 

### 2.3. Data Analysis

Data were exported from the ICPcovid platform, cleaned, and subsequently imported in R version 4.0.3 (R studio, Boston, United States of America) for analysis. Descriptive statistics are presented as means with standard deviation (SD) or medians with interquartile range (IQR) for continuous variables, and percentages (%) for categorical variables. Student t-tests and Mann–Whitney U tests were used to compare continuous variables between groups as appropriate. A chi square test was used to compare categorical variables between groups. Multivariable logistic regression modelling was used to assess factors associated with anxiety and depression and COVID-19 positivity. Potential contributing factors were evaluated using bivariate analysis (shown as crude OR). If more than two options were available for a categorical variable, the variable was dichotomized prior to regression analyses. Factors with a *p* < 0.250 in bivariate analysis were subsequently included in multivariate models. All statistical tests were two-sided. A *p*-value < 0.05 was considered statistically significant. 

## 3. Results

### 3.1. Respondent Characteristics

A total of 295 Brazilian HCW completed the online survey. Participants were from all macro-regions of the country. The mean age was 44 ± 12 years; 78.0% were women (Table 1). Ninety-five (32.2%) were medical doctors and 53 (18.0%) were nurses. Two-thirds (67.1%) of the HCW were working for government institutions. In total, 183 (62.0%) were directly involved in patient care.

### 3.2. Impact of COVID-19 on Work Routine

Respectively, 138 (46.8%) and 48 (16.3%) of the HCW reported working on average nine hours or more daily, and 6 days or more weekly during the two weeks prior to participating in the survey [9]. COVID-19 related restructuring at the health facilities was reported by 207 participants (70.2%). Sixty-nine (23.4%) HCW reported that their tasks changed due to COVID-19, 27 (39.1%) of whom indicated a relocation to a COVID-19 ward, 8 (11.6%) to an Intensive Care Unit (ICU), and 11 (15.9%) to an emergency department. Fifty (16.9%) HCW reported a decrease in salary during the COVID-19 pandemic, with a complete loss of salary reported by 5 (1.7%). HCW with a decrease in or loss of salary were older (51 ± 12 vs. 43 ± 12 years; *p* < 0.001), more frequently medical doctors (52.0% vs. 28.2%; *p* = 0.003), and showed a trend towards reporting less frequently close contact (<2 m distance) with a COVID-19-suspected patient (48.1% vs. 36.0%; *p* = 0.115).

### 3.3. COVID-19 Preventive and Protective Behavior

**Figure 1:**
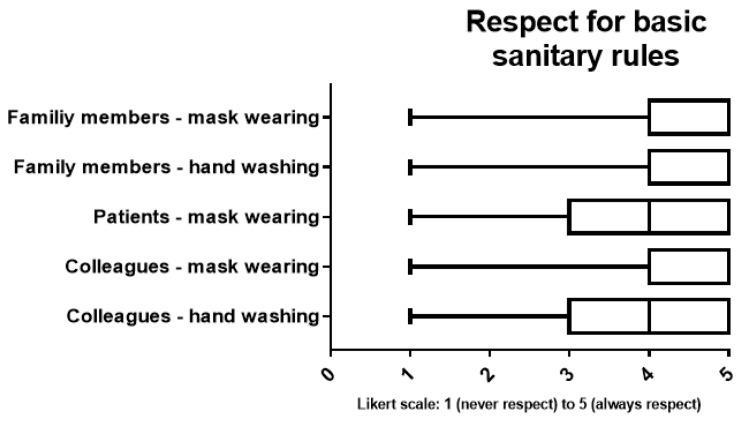
Boxplot representations of the participants’ perception of respect for hygienic measures by the entourage.

Nearly all HCW (95.9%) reported always wearing a face mask when going outside of their houses; only 2 (0.7%) reported never wearing a face mask and 5 (1.7%) reported to only wear a mask in the hospital. A total of 228 (77.8%) respondents indicated that they changed their mask at least daily. Most of the respondents reported to have the feeling that their entourage respected the basic sanitary rules very well (Figure 1).

A total of 136 HCW indicated having had close contact (<2 m distance) with COVID-19-suspected patients. Of these, 130 (95.6%) washed their hands or used hand sanitizer between each patient; 116/136 (85.3%) were wearing a protective apron when seeing the patient; 134/136 (98.5%) wore protective glasses or a face shield; 134/136 (98.5%) were wearing a mask, 87 of whom (64.9%) changed their mask at least daily. Of the 36 HCW who were asked in a follow-up questionnaire whether they were willing to be vaccinated with a COVID-19 vaccine, 33 (92%) answered that they would be willing to be vaccinated, including with a Chinese vaccine. 

### 3.4. Flu-Like Symptoms and COVID-19 Testing among HCW in Brazil

**Figure 2:**
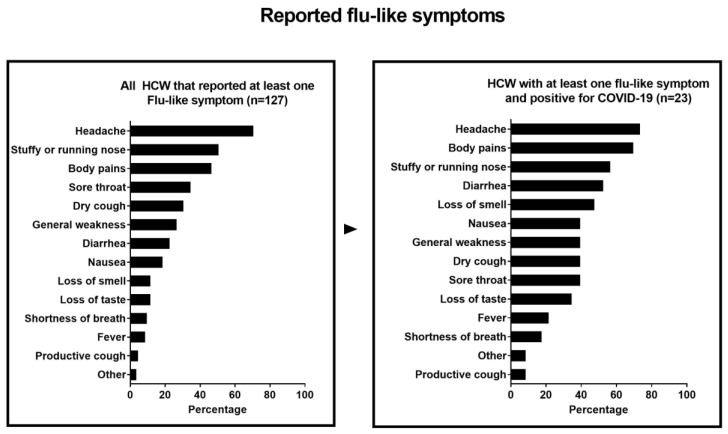
Flu-like symptoms among Health Care Workers in Brazil. HCW: Health Care Workers.

Overall, 127 (43.1%) HCW reported to have experienced at least one flu-like symptom since the onset of the pandemic. The most frequently reported symptom was headache (90/127; 70.9%), followed by stuffy or running nose (64/127; 50.4%) and general body pains (59/127; 46.5%) (Figure 2). Of those with flu-like symptoms, 83/127 (65.4%) fulfilled the clinical case definition for a suspected COVID-19 infection, 28 (33.7%) of whom reported no close contact with a COVID-19-suspected patient [26]. In mulivariable regression analyses, HCW who were directly involved in patient care were more likely to have experienced a flu-like illness fulfilling the COVID-19 clinical case definition criteria (OR = 1.91; 95% CI: 1.04–3.51) (Table 1). Married or cohabitating HCW (OR = 0.38; 95% CI: 0.22–0.67) and HCW working in hospitals that had been restructured due to COVID were less likely to have experienced a flu-like illness fulfilling the COVID-19 clinical case definition criteria (OR = 0.45; 95% CI: 0.24–0.82) (Table 2).

COVID-19 testing was performed in 217 (73.6%) respondents; 49 (22.6%) of them had at least one positive COVID-19 test. Only 23/49 (46.9%) HCW with a positive COVID-19 test reported one or more flu-like symptoms, suggesting that the infection was asymptomatic in 26/49 (53.1%). Of the 49 HCW with a positive COVID-19 test, 21 (42.8%) reported no close contact (<2 m distance) with a COVID-19 suspected patient. Following a positive COVID-19 test, 45/49 (91.8%) stopped working and stayed home, and 4/49 (8.2%) continued work. Two HCW required hospitalization for COVID-19. The only factor associated with a positive COVID-19 test in multivariable analysis was the number of flu-like symptoms experienced (OR = 1.17; 95% CI: 1.06–1.29; Supplementary Table S1).

### 3.5. Psychosocial Well-Being

More than half of the HCW (167; 56.6%) screened positive for anxiety (HADS-A ≥ 8) and 137 (46.4%) for depression (HADS-D ≥ 8). Approximately one-third (109; 36.9%) screened positive for both anxiety and depression. 

Female gender (OR = 2.40; 95% CI: 1.31–4.38), number of flu-like symptoms (OR = 1.25; 95% CI: 1.11–1.42) and being a nurse, medical doctor, or lab technician (OR = 1.69; 95% CI: 1.02–2.85) increased the odds for anxiety (Table 3). 

Female gender (OR = 2.41; 95% CI: 1.31–4.44) and number of flu-like symptoms (OR = 1.21; 95% CI: 1.08–1.35) also increased the odds of screening positive for depression (Table 4).

## 4. Discussion

With this online survey among Brazilian HCW, we found that the COVID-19 pandemic had an important impact on their work routines, with a high incidence of COVID-19 infections despite an effective implementation of COVID-19 preventive and protective measures. Almost three-quarters of the HCW reported a structural change in their health facilities to better accommodate patients, one-quarter reported a relocation to another hospital service, and one-sixth of the HCW reported a decrease in salary. This all coincided with high rates of anxiety and depression. Taken together, our findings point towards a massive impact of the COVID-19 pandemic, not only on HCW but also on patient care, with a wave of complications related to the postponement of non-COVID-19 care to be expected, as suggested by several studies [27,28,29,30,31].

Nearly all our respondents (>95%) reported always wearing a face mask when going outside. In modelling studies, universal mask use was shown to be highly effective in preventing a COVID-19 epidemic [32,33,34]. However, only three-fourths indicated that they changed their face mask daily. Whether this affects the effectiveness of mask wearing for COVID-19 prevention remains to be investigated. When caring for suspected COVID-19 patients, additional measures such as hand washing, and wearing a protective apron and face shield were also respected by almost all HCW. 

Despite good self-reported adherence to COVID-19 preventive and protective measures, we noticed a high COVID-19 positivity rate among HCW. Approximately one-third of the respondents in this study fulfilled the criteria of the clinical definition of COVID-19, and one-sixth reported at least one positive COVID-19 test. Studies in western countries also reported an increased prevalence of COVID-19 infection in HCW despite good availability of PPE and adherence to preventive and protective measures [35,36,37]. The reasons behind this observation warrant further investigation. It is possible that incorrect re-use of PPE as was reported in Brazil increased the risk of COVID-19 seropositivity [9,38,39]. One may also speculate that non-suspected, asymptomatic or pre-symptomatic patients could be an important source of transmission [40,41,42,43]. Moreover, given the high rate of community COVID-19 transmission in Brazil, many HCW may also have been contaminated outside the workplace [15,16,17]. In a study conducted between March and June 2020 among HCW in Sao Paulo, Brazil, the use of public transport to commute between home and the hospital was associated with an increased chance of COVID-19 seropositivity [18]. Finally, it is important to note that the adherence to preventive measures in our study was self-reported without a possibility to verify the reliability of this information. 

Unfortunately, in the current study, because of the small sample size, we could not identify relevant risk factors for COVID-19 positivity. However, HCW who were directly involved in patient care were more likely to have experienced a flu-like illness fulfilling the WHO COVID-19 clinical case definition. In contrast, HCW working in a hospital that was restructured for COVID-19 care were less likely to have experienced a flu-like illness fulfilling the COVID-19 clinical case definition criteria. In a systematic review of 97 studies from Europe (31), the United States, (9) and Asia (6), including 230,398 HCW, the estimated prevalence of SARS-CoV-2 infection based on RT-PCR results was 11% (95% CI; 7–15%). Nurses were the most frequently affected HCW (48%), and most of the COVID-19 positive medical personnel were working in hospitalization/non-emergency wards [37]. This systematic review thus also confirms that HCW with direct patient contact face the highest risk of COVID-19 positivity.

Despite that the question about willingness to be vaccinated was asked to a small number of HCW, COVID-19 vaccine acceptance was high (92%). This corresponds with the results of a more recent online survey among the general population in Brazil. Of the 6470 participants in this survey, 94.2% responded to be willing to be COVID-19 vaccinated if the vaccine was 95% effective, and 88.9% if the vaccine was 90% effective [44]. This high acceptance rate of the COVID-19 vaccine is surprising given the negative attitude of the Brazilian president regarding COVID-19 vaccination.

During the COVID-19 pandemic, HCW experience acute stress leading to emotional, cognitive, physical and relationship problems [45]. A recent umbrella systematic review reported that one-third of HCW manifested signs of burnout syndrome [46]. The stressful work environment, the long working hours leading to fatigue, and the psychological problems related to isolation also contributed to the increased risk of becoming infected with COVID-19 [47,48].

A cross-sectional survey based on the National Internet Survey on Emotional and Mental Health (NISEMH), China, found that, compared to the general population, the mental health of HCW may indeed be more severely impacted. The overall prevalence of generalized anxiety disorder (GAD), depressive symptoms, and sleep quality stratified by gender, age, and occupations were 35.1%, 20.1%, and 18.2%, respectively. The prevalence of GAD and depressive symptoms was significantly higher in participants younger than 35 years than in participants aged 35 years or older. Compared with other occupational groups, HCW (23.6%) reported the highest rate of poor sleep quality [49]. Within the included HCW, a pooled analysis of 33,062 participants revealed gender and occupational differences with female HCW and nurses exhibiting higher rates of affective symptoms compared to male and medical staff, respectively. The latter is in line with the findings from our study, where the female gender was associated with a higher odd for anxiety as well as for depression. Being a medical doctor, nurse, or lab technician was associated with a higher odd for anxiety [50]. In another recent analysis incorporating 65 studies with a total of 97,333 HCW, the pooled prevalence of depression and anxiety during the COVID-19 pandemic was 21.7% and 22.1%, respectively [6].

In our study, approximately half of the respondents screened positive for anxiety, half screened positive for depression, and one-third screened positive for both conditions, suggesting an important psychosocial burden among HCW caused by the ongoing pandemic. These findings indicate the worsening of the mental health condition of HCW during the pandemic, suggesting that interventions need to be considered to rescue the quality of life of HCW during and after the pandemic.

Several limitations of our study must be acknowledged. First, the study was based on an online survey with an inherent risk for selection bias. Secondly, it was difficult to estimate the increase in workload with the current design of our questionnaire. In addition, the questionnaire we used prohibited us from drawing a firm conclusion regarding the causal effect between different preventive measures for COVID-19 and the incidence of COVID-19. Thirdly, the sample size was limited and therefore detailed subgroup analysis was not possible. The questionnaire was distributed at a moment when Brazil was facing a high number of new COVID-19 cases and deaths. HCW were overwhelmed with work and therefore may have been too tired to participate in the survey. Lastly, we did not ask questions regarding the use of PPE to respondents who reported no close contact (<2 m) with suspected patients. As such, we cannot exclude that COVID-19 transmission occurred when caring for non-suspected COVID-19 patients or as a consequence of community transmission outside the health care setting, which may possibly explain the high incidence of COVID-19 noted in this study. Among the HCW that responded to the clinical definition or had a positive COVID-19 test, approximately 40% reported no close contact with COVID-19 suspected patients. Further studies are needed to investigate the underlying causes of the high COVID-19 incidence among Brazilian HCW.

Despite all these limitations, our study highlights the urgent need for interventions to mitigate the psychosocial risks HCW in Brazil encounter while facing the consecutive waves of the COVID-19 pandemic. This requires a multi-dimensional approach that needs to include practical support such as providing safe areas for rest and relaxation at the workplace, accommodation solutions for HCW who cannot stay at their home, offering free meals, and childcare services. Sufficient recovering time is essential. Psychological support should be offered inside the healthcare settings, with the possibility of complementary support through a buddy system, informal support groups, a psychological hotline and/or other forms of telemedicine [51]. The way the health facility is managed is also important to reduce the stress among HCW. Consultation and engagement with HCW is key. The management should listen to them and address their safety, financial, organizational, and other concerns. The work of the HCW should be valued, and they should be remunerated appropriately. They should feel supported by society and the authorities.

## 5. Conclusions

In conclusion, despite a limited sample size, our study suggests that COVID-19 had an important impact on Brazilian HCW with a high incidence of COVID-19 infections despite adherence to preventive COVID-19 measures and high rates of anxiety and depression. Targeted interventions to reduce the rates of COVID-19 infection, as well as anxiety and depression, should be developed in order to keep HCW healthy and at work for as long as possible. Promoting universal PPE use during patient care and promoting vaccination may be ideas in this regard.

## Figures and Tables

**Table 1:** Overview of the respondents’ characteristics.

Characteristics		Total *n* = 295
Demographics	Age: Mean ± SD	44 ± 12 years
	Gender: n (%)	
	Female	230 (78.0%)
	Male	65 (22.0%)
	Religion: n (%)	
	Catholic	130 (44.1%)
	Pentecostal	6 (2.0%)
	Protestant	36 (11.9%)
	Other	75 (24.8%)
	None	52 (17.2%)
	Marital status: n (%)	
	Single	75 (25.4%)
	In a relationship but not cohabitating	24 (8.1%)
	Cohabitation/Legally married	155 (52.5%)
	Divorced	34 (11.5%)
	Widow/Other	7 (2.4%)
Profession	Task description: n (%)	
	Nurse	53 (18.0%)
	Laboratory staff	27 (9.2%)
	Medical doctor	95 (32.2%)
	Administrative staff	82 (27.8%)
	Other	38 (12.8%)
	Organization type: n (%)	
	Government	198 (67.1%)
	Private	83 (28.1%)
	Other	14 (4.7%)
	Directly involved in patient care: n (%)	183 (62.0%)
	In an internal medicine ward	44/183 (24.0%)
	In another ward	25/183 (13.7%)
	In an intensive care unit (ICU)	38/183 (20.8%)
	In an emergency department	23/183 (12.6%)
	In an outpatient department	67/183 (36.6%)
	Other	62/183 (33.9%)

**Table 2:** Multiple logistic regression model for factors associated with fulfilling the WHO clinical definition of COVID-19.

Covariates	Clinical Definition Negative (*n* = 212)	Clinical Definition Positive (*n* = 83)	Crude OR (95% CI)	Adjusted OR (95% CI)	*p*-Value
Gender					0.657
Male	46 (21.7%)	19 (22.9%)	Ref	Ref	
Female	166 (78.3%)	64 (77.1%)	0.93 (0.51–1.71)	0.86 (0.44–1.67)	
Hospital Restructured Due to COVID					0.009
No	54 (25.5%)	34 (41%)	Ref	Ref	
Yes	158 (74.5%)	49 (59%)	0.49 (0.29–0.84)	0.45 (0.24–0.82)	
Directly involved in patient care					0.036
No	86 (40.6%)	26 (31.3%)	Ref	Ref	
Yes	126 (59.4%)	57 (68.7)	1.5 (0.87–2.56)	1.91 (1.04–3.51)	
Marital status					0.001
Single/divorced	87 (41%)	53 (64%)	Ref	Ref	
Married/cohabitating	125 (59%)	30 (36%)	0.39 (0.23–0.67)	0.38 (0.22–0.67)	

OR: Odds ratio; CI: Confidence interval; Ref: Reference category; IQR: Interquartile range.

**Table 3:** Multiple logistic regression model for factors associated with anxiety (HADS-A ≥ 8).

Covariates	No Anxiety (*n* = 128)	Anxiety (*n* = 167)	Crude OR (95% CI)	Adjusted OR (95% CI)	*p*-Value
Gender					0.004
Male	38 (29.7%)	27 (16.2%)	Ref	Ref	
Female	90 (70.3%)	140 (83.8%)	2.19 (1.25–3.83)	2.40 (1.31–4.38)	
Age: Median (IQR)	49 (35–56)	40 (33–52)	0.97 (0.95–0.99)	0.97 (0.95–0.99)	0.043
Hospital restructured due to COVID-19					0.027
No	30 (23.4%)	58 (34.7%)	Ref	Ref	
Yes	98 (76.6%)	109 (65.3%)	0.58 (0.34–0.97)	0.53 (0.30–0.93)	
Number of flu-like symptoms: Median (IQR)	0 (0–1)	0 (0–3)	1.28 (1.13–1.44)	1.25 (1.11–1.42)	<0.001
Profession					0.046
Administrative staff, others	56 (43.8%)	64 (38.3%)	Ref	Ref	
Nurse, medical doctor, lab technician	72 (56.2%)	103 (61.7%)	1.25 (0.78–2)	1.69 (1.02–2.85)	

OR: Odds ratio; CI: Confidence interval; Ref: Reference category; IQR: Interquartile range.

**Table 4:** Multiple logistic regression model for factors associated with depression (HADS-D ≥ 8).

Covariates	No Depression (*n* = 158)	Depression (*n* = 137)	Crude OR (95% CI)	Adjusted OR (95% CI)	*p*-Value
Gender					0.004
Male	45 (28.5%)	20 (14.6%)	Ref	Ref	
Female	113 (71.5%)	117 (85.4%)	2.33 (1.30–4.19)	2.41 (1.31–4.44)	
Age: Median (IQR)	46 (33–55)	42 (34–53)	0.99 (0.97–1.01)	0.99 (0.98–10.2)	0.877
Hospital restructured due to COVID-19					0.072
No	39 (24.7%)	49 (35.8%)	Ref	Ref	
Yes	119 (75.3%)	88 (64.2%)	0.59 (0.36–0.97)	0.61 (0.35–1.05)	
Number of flu-like symptoms: Median (IQR)	0 (0–2)	0 (0–3)	1.22 (1.10–1.35)	1.21 (1.08–1.35)	<0.001
Profession					0.715
Administrative staff, others	62 (39%)	58 (42.3%)	Ref	Ref	
Nurse, medical doctor, lab technician	96 (61%)	79 (57.7%)	0.88 (0.55–1.40)	1.09 (0.66–1.81)	

OR: Odds ratio; CI: Confidence interval; Ref: Reference category; IQR: Interquartile range.

## Data Availability

The data presented in this study are available on request from the corresponding author.

## Supplementary Materials

The following are available online at https://www.mdpi.com/article/10.3390/ijerph18126511/s1. Table S1: Multivariate logistic regression model for factors associated with a COVID-19 positive test. Table S2: Questionnaire survey. Table S3: Follow-up survey.
