# Supplementary material for: Impact of COVID-19 on Healthcare Workers in Brazil between August and November 2020: A Cross-Sectional Survey
Source: Int J Environ Res Public Health. 2021 Jun 17;18(12):6511. doi: 10.3390/ijerph18126511 (PMC8296453; doi:10.3390/ijerph18126511)
Supplement: Supplementary file 1 [file ijerph-18-06511-s001.zip › ijerph-1258440-supplementary/ijerph-1258440-tableS2.pdf]

Covid-19 Questionnaire for Healthcare Workers

General questions

THIS QUESTIONNAIRE IS STRICTLY RESERVED TO HEALTHCARE PERSONNEL AND OTHER PERSONS WORKING IN THE HEALTH SECTOR. WE SEEK TO UNDERSTAND HOW THEY HAVE BEEN AFFECTED BY THE ONGOING CORONAVIRUS PANDEMIC. THANK YOU FOR YOUR PARTICIPATION.

Q: Please confirm that you are a healthcare personnel or someone actively working in the healthcare sector(*)

Type: choice

A: one of the following:

yes	=>	*Yes, I confirm that I work in healthcare*
no	=>	*No, I am not involved in the health sector*

Q: Age (in years) (*)

Type: number

A: number (min: 1 / max: 110 / step: 1)

Q: Gender (*)

Type: choice

A: one of the following:

male	=>	*Male*
female	=>	*Female*

Q: Religion (*)

Type: choice

A: one of the following:

catholic	=>	*Christian: Catholic*
protestant	=>	*Christian: Protestant*
muslim	=>	*Muslim*
other	=>	*Other*
none	=>	*None*

Q: Marital status (*)

Type: choice

A: one of the following:

single	=>	*Single*
cohabit	=>	*Cohabitation*
legally_married	=>	*Legally married*
divorced	=>	*Divorced*

widow_widower	=>	*Widow/widower*
not_married_not_cohab_but_in_relationship	=>	*I am not married, nor in cohabitation, but I am in a relationship*

Q: Which country do you currently live in? (*)

Type: choice

A: one of the following:

Thailand	=>	*Thailand*
Afghanistan	=>	*Afghanistan*
Albania	=>	*Albania*
Algeria	=>	*Algeria*
American Samoa	=>	*American Samoa*
Andorra	=>	*Andorra*
Angola	=>	*Angola*
Anguilla	=>	*Anguilla*
Antarctica	=>	*Antarctica*
Antigua & Barbuda	=>	*Antigua & Barbuda*
Argentina	=>	*Argentina*
Armenia	=>	*Armenia*
Aruba	=>	*Aruba*
Ascension Island	=>	*Ascension Island*
Australia	=>	*Australia*
Austria	=>	*Austria*
Azerbaijan	=>	*Azerbaijan*
Bahamas	=>	*Bahamas*
Bahrain	=>	*Bahrain*
Bangladesh	=>	*Bangladesh*
Barbados	=>	*Barbados*
Belarus	=>	*Belarus*
Belgium	=>	*Belgium*
Belize	=>	*Belize*
Benin	=>	*Benin*
Bermuda	=>	*Bermuda*
Bhutan	=>	*Bhutan*
Bolivia	=>	*Bolivia*
Bosnia & Herzegovina	=>	*Bosnia & Herzegovina*
Botswana	=>	*Botswana*
Bouvet Island	=>	*Bouvet Island*
Brazil	=>	*Brazil*
British Indian Ocean Territory	=>	*British Indian Ocean Territory*
British Virgin Islands	=>	*British Virgin Islands*
Brunei	=>	*Brunei*
Bulgaria	=>	*Bulgaria*
Burkina Faso	=>	*Burkina Faso*
Burundi	=>	*Burundi*

Cambodia	=>	*Cambodia*
Cameroon	=>	*Cameroon*
Canada	=>	*Canada*
Canary Islands	=>	*Canary Islands*
Cape Verde	=>	*Cape Verde*
Caribbean Netherlands	=>	*Caribbean Netherlands*
Cayman Islands	=>	*Cayman Islands*
Central African Republic	=>	*Central African Republic*
Ceuta & Melilla	=>	*Ceuta & Melilla*
Chad	=>	*Chad*
Chile	=>	*Chile*
China	=>	*China*
Christmas Island	=>	*Christmas Island*
Clipperton Island	=>	*Clipperton Island*
Cocos (Keeling) Islands	=>	*Cocos (Keeling) Islands*
Colombia	=>	*Colombia*
Comoros	=>	*Comoros*
Congo - Brazzaville	=>	*Congo - Brazzaville*
Congo - Kinshasa	=>	*Congo - Kinshasa*
Cook Islands	=>	*Cook Islands*
Costa Rica	=>	*Costa Rica*
Croatia	=>	*Croatia*
Cuba	=>	*Cuba*
Curaçao	=>	*Curaçao*
Cyprus	=>	*Cyprus*
Czechia	=>	*Czechia*
Côte d’Ivoire	=>	*Côte d’Ivoire*
Denmark	=>	*Denmark*
Diego Garcia	=>	*Diego Garcia*
Djibouti	=>	*Djibouti*
Dominica	=>	*Dominica*
Dominican Republic	=>	*Dominican Republic*
Ecuador	=>	*Ecuador*
Egypt	=>	*Egypt*
El Salvador	=>	*El Salvador*
Equatorial Guinea	=>	*Equatorial Guinea*
Eritrea	=>	*Eritrea*
Estonia	=>	*Estonia*
Eswatini	=>	*Eswatini*
Ethiopia	=>	*Ethiopia*
Falkland Islands	=>	*Falkland Islands*
Faroe Islands	=>	*Faroe Islands*
Fiji	=>	*Fiji*
Finland	=>	*Finland*
France	=>	*France*
French Guiana	=>	*French Guiana*

French Polynesia	=>	*French Polynesia*
French Southern Territories	=>	*French Southern Territories*
Gabon	=>	*Gabon*
Gambia	=>	*Gambia*
Georgia	=>	*Georgia*
Germany	=>	*Germany*
Ghana	=>	*Ghana*
Gibraltar	=>	*Gibraltar*
Greece	=>	*Greece*
Greenland	=>	*Greenland*
Grenada	=>	*Grenada*
Guadeloupe	=>	*Guadeloupe*
Guam	=>	*Guam*
Guatemala	=>	*Guatemala*
Guernsey	=>	*Guernsey*
Guinea	=>	*Guinea*
Guinea-Bissau	=>	*Guinea-Bissau*
Guyana	=>	*Guyana*
Haiti	=>	*Haiti*
Heard & McDonald Islands	=>	*Heard & McDonald Islands*
Honduras	=>	*Honduras*
Hong Kong SAR China	=>	*Hong Kong SAR China*
Hungary	=>	*Hungary*
Iceland	=>	*Iceland*
India	=>	*India*
Indonesia	=>	*Indonesia*
Iran	=>	*Iran*
Iraq	=>	*Iraq*
Ireland	=>	*Ireland*
Isle of Man	=>	*Isle of Man*
Israel	=>	*Israel*
Italy	=>	*Italy*
Jamaica	=>	*Jamaica*
Japan	=>	*Japan*
Jersey	=>	*Jersey*
Jordan	=>	*Jordan*
Kazakhstan	=>	*Kazakhstan*
Kenya	=>	*Kenya*
Kiribati	=>	*Kiribati*
Kosovo	=>	*Kosovo*
Kuwait	=>	*Kuwait*
Kyrgyzstan	=>	*Kyrgyzstan*
Laos	=>	*Laos*
Latvia	=>	*Latvia*
Lebanon	=>	*Lebanon*
Lesotho	=>	*Lesotho*

Liberia	=>	*Liberia*
Libya	=>	*Libya*
Liechtenstein	=>	*Liechtenstein*
Lithuania	=>	*Lithuania*
Luxembourg	=>	*Luxembourg*
Macao SAR China	=>	*Macao SAR China*
Madagascar	=>	*Madagascar*
Malawi	=>	*Malawi*
Malaysia	=>	*Malaysia*
Maldives	=>	*Maldives*
Mali	=>	*Mali*
Malta	=>	*Malta*
Marshall Islands	=>	*Marshall Islands*
Martinique	=>	*Martinique*
Mauritania	=>	*Mauritania*
Mauritius	=>	*Mauritius*
Mayotte	=>	*Mayotte*
Mexico	=>	*Mexico*
Micronesia	=>	*Micronesia*
Moldova	=>	*Moldova*
Monaco	=>	*Monaco*
Mongolia	=>	*Mongolia*
Montenegro	=>	*Montenegro*
Montserrat	=>	*Montserrat*
Morocco	=>	*Morocco*
Mozambique	=>	*Mozambique*
Myanmar (Burma)	=>	*Myanmar (Burma)*
Namibia	=>	*Namibia*
Nauru	=>	*Nauru*
Nepal	=>	*Nepal*
Netherlands	=>	*Netherlands*
Netherlands Antilles	=>	*Netherlands Antilles*
New Caledonia	=>	*New Caledonia*
New Zealand	=>	*New Zealand*
Nicaragua	=>	*Nicaragua*
Niger	=>	*Niger*
Nigeria	=>	*Nigeria*
Niue	=>	*Niue*
Norfolk Island	=>	*Norfolk Island*
Northern Mariana Islands	=>	*Northern Mariana Islands*
North Korea	=>	*North Korea*
North Macedonia	=>	*North Macedonia*
Norway	=>	*Norway*
Oman	=>	*Oman*
Outlying Oceania	=>	*Outlying Oceania*
Pakistan	=>	*Pakistan*

Palau	=>	*Palau*
Palestinian Territories	=>	*Palestinian Territories*
Panama	=>	*Panama*
Papua New Guinea	=>	*Papua New Guinea*
Paraguay	=>	*Paraguay*
Peru	=>	*Peru*
Philippines	=>	*Philippines*
Pitcairn Islands	=>	*Pitcairn Islands*
Poland	=>	*Poland*
Portugal	=>	*Portugal*
Puerto Rico	=>	*Puerto Rico*
Qatar	=>	*Qatar*
Romania	=>	*Romania*
Russia	=>	*Russia*
Rwanda	=>	*Rwanda*
Réunion	=>	*Réunion*
Samoa	=>	*Samoa*
San Marino	=>	*San Marino*
Saudi Arabia	=>	*Saudi Arabia*
Senegal	=>	*Senegal*
Serbia	=>	*Serbia*
Seychelles	=>	*Seychelles*
Sierra Leone	=>	*Sierra Leone*
Singapore	=>	*Singapore*
Sint Maarten	=>	*Sint Maarten*
Slovakia	=>	*Slovakia*
Slovenia	=>	*Slovenia*
Solomon Islands	=>	*Solomon Islands*
Somalia	=>	*Somalia*
South Africa	=>	*South Africa*
South Georgia & South Sandwich Islands	=>	*South Georgia & South Sandwich Islands*
South Korea	=>	*South Korea*
South Sudan	=>	*South Sudan*
Spain	=>	*Spain*
Sri Lanka	=>	*Sri Lanka*
St. Barthélemy	=>	*St. Barthélemy*
St. Helena	=>	*St. Helena*
St. Kitts & Nevis	=>	*St. Kitts & Nevis*
St. Lucia	=>	*St. Lucia*
St. Martin	=>	*St. Martin*
St. Pierre & Miquelon	=>	*St. Pierre & Miquelon*
St. Vincent & Grenadines	=>	*St. Vincent & Grenadines*
Sudan	=>	*Sudan*
Suriname	=>	*Suriname*
Svalbard & Jan Mayen	=>	*Svalbard & Jan Mayen*

Sweden	=>	*Sweden*
Switzerland	=>	*Switzerland*
Syria	=>	*Syria*
São Tomé & Príncipe	=>	*São Tomé & Príncipe*
Taiwan	=>	*Taiwan*
Tajikistan	=>	*Tajikistan*
Tanzania	=>	*Tanzania*
Timor-Leste	=>	*Timor-Leste*
Togo	=>	*Togo*
Tokelau	=>	*Tokelau*
Tonga	=>	*Tonga*
Trinidad & Tobago	=>	*Trinidad & Tobago*
Tristan da Cunha	=>	*Tristan da Cunha*
Tunisia	=>	*Tunisia*
Turkey	=>	*Turkey*
Turkmenistan	=>	*Turkmenistan*
Turks & Caicos Islands	=>	*Turks & Caicos Islands*
Tuvalu	=>	*Tuvalu*
U.S. Outlying Islands	=>	*U.S. Outlying Islands*
U.S. Virgin Islands	=>	*U.S. Virgin Islands*
Uganda	=>	*Uganda*
Ukraine	=>	*Ukraine*
United Arab Emirates	=>	*United Arab Emirates*
United Kingdom	=>	*United Kingdom*
United States	=>	*United States*
Uruguay	=>	*Uruguay*
Uzbekistan	=>	*Uzbekistan*
Vanuatu	=>	*Vanuatu*
Vatican City	=>	*Vatican City*
Venezuela	=>	*Venezuela*
Vietnam	=>	*Vietnam*
Wallis & Futuna	=>	*Wallis & Futuna*
Western Sahara	=>	*Western Sahara*
Yemen	=>	*Yemen*
Zambia	=>	*Zambia*
Zimbabwe	=>	*Zimbabwe*
Åland Islands	=>	*Åland Islands*

Q: In which state of Brazil do you live? (*)

Type: choice

A: one of the following:

Acre	=>	*Acre*
Alagoas	=>	*Alagoas*
Amapá	=>	*Amapá*
Amazonas	=>	*Amazonas*

Bahia	=>	*Bahia*
Ceará	=>	*Ceará*
Distrito Federal	=>	*Distrito Federal*
Espírito Santo	=>	*Espírito Santo*
Goiás	=>	*Goiás*
Maranhão	=>	*Maranhão*
Mato Grosso	=>	*Mato Grosso*
Mato Grosso do Sul	=>	*Mato Grosso do Sul*
Minas Gerais	=>	*Minas Gerais*
Pará	=>	*Pará*
Paraíba	=>	*Paraíba*
Paraná	=>	*Paraná*
Pernambuco	=>	*Pernambuco*
Piauí	=>	*Piauí*
Rio de Janeiro	=>	*Rio de Janeiro*
Rio Grande do Norte	=>	*Rio Grande do Norte*
Rio Grande do Sul	=>	*Rio Grande do Sul*
Rondônia	=>	*Rondônia*
Roraima	=>	*Roraima*
Santa Catarina	=>	*Santa Catarina*
São Paulo	=>	*São Paulo*
Sergipe	=>	*Sergipe*
Tocantins	=>	*Tocantins*

Visible if

Q:	A:
0	- :input[name="which_country_do_you_currently_live_in_"] =>
1	
2	- :input[name="which_country_do_you_currently_live_in_"] =>

Q: Which city/village do you live in? (*)

Type: text

A: text input

Q: Which organisation do you work for? (*)

Type: choice

A: one of the following:

government	=>	*Public (government)*
private	=>	*Private (individual or NGO)*
other	=>	*Other*

Q: Which hospital or other health structure are you working in? (*)

Type: text

A: text input

Q: According to you, what is the current stage of the COVID-19 epidemic in the area where you live?(*)

Type: choice

A: one of the following:

initial	=>	*Initial stage, with very little transmission of COVID-19*
ongoing	=>	*Stage of high ongoing transmission of COVID-19*
decline	=>	*Stage of declining COVID-19 transmission following a peak period*
second_wave	=>	*Second wave of COVID-19 with increasing cases*

Q: What is your profession? (*)

Type: choice

A: one of the following:

chw	=>	*Community Health Worker*
nurse	=>	*Nurse*
clinical_officer	=>	*Clinical Officer*
laboratory	=>	*Laboratory staff*
pharmacy	=>	*Pharmacy staff*
generalist	=>	*Medical doctor: general practitioner*
resident	=>	*Medical doctor: Intern or resident*
specialist	=>	*Medical doctor: specialist*
administrative	=>	*Administrative person working in a hospital or clinic*
support_staff	=>	*Support staff working in a hospital or clinic (driver, cleaner, maintenance, etc)*
other	=>	*Other*

Q: What is your medical specialty?

Type: text

A: text input

Visible if

Q:	A:
0	- :input[name="what_is_your_profession_"] =>
1	
2	- :input[name="what_is_your_profession_"] =>

Q: Are you directly involved in patient care? (*)

Type: choice

A: one of the following:

yes	=>	*Yes/ Haa*
no	=>	*No/ Maya*

Q: If yes, in which service do you work? (multiple answers possible) (*)

Type: choice_multiple

A: multiple answers possible:

hospitalisation	=>	*Patient hospitalisation ward/internal medicine ward*
other_ward	=>	*In another ward (surgery, pediatrics, obstetrics/gynaecology)*
icu	=>	*Intensive Care Unit (ICU)*
emergency	=>	*The emergency department*
outpatient	=>	*Outpatient clinic / department*
other	=>	*Other (specify)*

Visible if

Q:	A:
Are you directly involved in patient care?	- value => yes

Q: (OPTIONAL) Specify other service/department where you work, even outside the hospital:

Type: text

A: text input

Q: What are your lodging/accommodation conditions? (*)

Type: choice

A: one of the following:

hospital	=>	*I live on the hospital premises*
beside_hospital	=>	*I live in lodging facilities very close to the hospital*
house	=>	*I live in a home away from the hospital*
another_town	=>	*I live in another town/village and I travel daily to work in the hospital*
other	=>	*Other (specify)*

Q: Specify other housing conditions: (*)

Type: text

A: text input

Visible if

Q:	A:
What are your lodging/accommodation conditions?	- value => other

Q: Who are you currently living with? (multiple answers possible) (*)

Type: choice_multiple

A: multiple answers possible:

parents	=>	*My parent(s)*
spouse	=>	*My spouse/partner*
children	=>	*My child(ren)*
siblings	=>	*My sibling(s) or other relative(s)*
friends	=>	*My friend(s)*
colleagues	=>	*My colleague(s)*
alone	=>	*I live alone*

Work-related questions

Q: During the last two weeks: How many days did you go to work each week? (*)

Type: number

A: number (min: n/a / max: 7 / step: n/a)

Q: During the last two weeks: How many hours (on average) did you spend at work each day? (*)

Type: number

A: number (min: n/a / max: 24 / step: n/a)

Q: Has your hospital been restructured to better accommodate COVID-19 patients? (*)

Type: choice

A: one of the following:

yes	=>	*Yes*
no	=>	*No*
do_not_know	=>	*I don't know*

Q: Have your tasks in the hospital changed because of the COVID-19 epidemic? (e.g. shifted to another ward or department, etc) (*)

Type: choice

A: one of the following:

yes	=>	*Yes*
no	=>	*No*
na	=>	*Not applicable*

Q: If yes, what was the change? (multiple answers possible) (*)

Type: choice_multiple

A: multiple answers possible:

covid_ward	=>	*I am now assisting in a ward with COVID patients*
icu	=>	*I am now assisting at the intensive care unit*
emergency	=>	*I am now assisting at the emergency department*
other	=>	*Other (specify)*

Visible if

Q:	A:
Have your tasks in the hospital changed because of the COVID-19 epidemic? (e.g. shifted to another ward or department, etc)	- value => yes

Q: Specify other changes at work:

Type: text

A: text input

Visible if

Q:	A:
:input[name="if_yes_what_was_the_change_[other]"]	- checked => 1

Q: Has your salary/income changed during the COVID-19 epidemic? (*)

Type: choice

A: one of the following:

yes	=>	*Yes*
no	=>	*No*
na	=>	*Not applicable*

Q: If yes: (*)

Type: choice

A: one of the following:

increased	=>	*I get a higher salary/income*
decreased	=>	*I get a lower salary/income, but I still get paid*
no_salary	=>	*They stopped paying my salary / I now have no income*

Visible if

Q:	A:
Has your salary/income changed during the COVID-19 epidemic?	- value => yes

Q: Have your housing conditions changed because of the coronavirus epidemic? (*)

Type: choice

A: one of the following:

yes_closer	=>	*Yes, I now live much closer to the hospital*
yes_away	=>	*Yes, I now live further away from the hospital*
no	=>	*No, nothing has changed concerning my housing conditions*

Q: Did any of your housemates relocate because of the coronavirus epidemic? (*)

Type: choice

A: one of the following:

yes	=>	*Yes*
no	=>	*No*
na	=>	*Not applicable*

Q: If yes, who are you living with now? (multiple answers possible) (*)

Type: choice_multiple

A: multiple answers possible:

parents	=>	*My parent(s)*
spouse	=>	*My spouse/partner*
children	=>	*My child(ren)*
siblings	=>	*My sibling(s) or other relative(s)*
friends	=>	*My friend(s)*
colleagues	=>	*My colleague(s)*
alone	=>	*I live alone*

Visible if

Q:	A:
Did any of your housemates relocate because of the coronavirus epidemic?	- value => yes

Q: Have you been using face masks for COVID-19 prevention? (*)

Type: choice

A: one of the following:

no	=>	*No, I do not wear face masks*
hospital_only	=>	*Yes, only in the hospital*
outside_occasionally	=>	*Yes, in the hospital and occasionally when I go outside (less than half of the times)*
outside_sometimes	=>	*Yes, in the hospital and sometimes when I go outside (more than half of the times)*
outside_always	=>	*Yes, in the hospital and anytime I go outside*

Q: If yes, which type of face masks do you use? (many answers possible) (*)

Type: choice_multiple

A: multiple answers possible:

surgical	=>	*Surgical masks*
cloth	=>	*Cloth masks*
N95_FFP2	=>	*N95, KN95, or FFP2 masks (respirators)*
other	=>	*Other types of mask*

Visible if

Q:	A:
Have you been using face masks for COVID-19 prevention?	- !value => no

Q: How often do you usually change your mask? (*)

Type: choice

A: one of the following:

never	=>	*Never*
once_week	=>	*1 time per week*
2_4_times_week	=>	*2-4 times per week*
5_6_times_week	=>	*5-6 times per week*
daily	=>	*Once a day*
multiple_daily	=>	*Multiple times a day*

Visible if

Q:	A:
Have you been using face masks for COVID-19 prevention?	- !value => no

Q: Have you been in close contact (<2 metres distance) with patients suspected to have COVID-19 in the hospital?(*)

Type: choice

A: one of the following:

yes	=>	*Yes*
no	=>	*No*
dont_know	=>	*I don't know*

Q: If yes, did you wash hands, or used hand sanitizer before and after you saw each patient?(*)

Type: choice

A: one of the following:

yes	=>	*Yes*
no_time	=>	*No: I wanted to, but there was no time to wash hands*
no_water_soap	=>	*No: I wanted to, but there was no water/soap/hand sanitizer*
no_forgot	=>	*No, I forgot to wash my hands*
no_not_necessary	=>	*No, I don’t think it is necessary to do this all the time*

Visible if

Q:	A:
Have you been in close contact (<2 metres distance) with patients suspected to have COVID-19 in the hospital?	- value => yes

Q: Did you wear a protective apron? (*)

Type: choice

A: one of the following:

yes_daily	=>	*Yes, and I changed my apron every day*
yes_not_daily	=>	*Yes, but I could not change my apron every day*
no_aprons	=>	*No, there were no aprons available*
no_not_necessary	=>	*No, I don't think it is necessary to wear an apron*

Visible if

Q:	A:
Have you been in close contact (<2 metres distance) with patients suspected to have COVID-19 in the hospital?	- value => yes

Q: Did you wear a mask? (*)

Type: choice

A: one of the following:

yes_daily	=>	*Yes, and I changed my mask every day*
yes_not_daily	=>	*Yes, but I could not change my mask every day*
no_masks	=>	*No, there were no masks available*
no_not_necessary	=>	*No, I didn't think it was necessary to wear a mask*

Visible if

Q:	A:
Have you been in close contact (<2 metres distance) with patients suspected to have COVID-19 in the hospital?	- value => yes

Q: Do you wear protective eye glasses or face shield when attending to patients? (*)

Type: choice

A: one of the following:

yes	=>	*Yes*
no_glasses	=>	*No, but I wear normal eyeglasses*
no	=>	*No, my eyes are unprotected*
na	=>	*Not applicable (I do not attend to patients)*

Q: How well do your colleagues respect the basic rules of regular hand washing, on a scale of 1 (do not at respect all) to 5 (always respect)? (*)

Type: choice

A: one of the following:

1	=>	*1*
2	=>	*2*
3	=>	*3*
4	=>	*4*
5	=>	*5*

Q: How well do your colleagues respect the basic rules of mask wearing, on a scale of 1 (do not respect at all) to 5 (always respect)? (*)

Type: choice

A: one of the following:

1	=>	*1*
2	=>	*2*
3	=>	*3*
4	=>	*4*
5	=>	*5*

Q: How well do the patients coming to consult at your hospital respect the basic rules of mask wearing, on a scale of 1 (do not respect at all) to 5 (always respect)? (*)

Type: choice

A: one of the following:

1	=>	*1*
2	=>	*2*
3	=>	*3*
4	=>	*4*
5	=>	*5*

Q: How well do your housemates/family members respect the basic rules of regular hand washing, on a scale of 1 (do not respect at all) to 5 (always respect)? (*)

Type: choice

A: one of the following:

1	=>	*1*
2	=>	*2*
3	=>	*3*
4	=>	*4*
5	=>	*5*

Q: How well do your housemates/family members respect the basic rules of mask wearing, on a scale of 1 (do not respect at all) to 5 (always respect)? (*)

Type: choice

A: one of the following:

1	=>	*1*
2	=>	*2*
3	=>	*3*
4	=>	*4*
5	=>	*5*

Health-related questions

Q: Since the beginning of the COVID-19 epidemic in your area, have you experienced any of the following flu-like symptoms? (multiple options possible) (*)

Type: choice_multiple

A: multiple answers possible:

fever	=>	*Fever*
headache	=>	*Headache*
sore throat	=>	*Sore throat*
loss_taste	=>	*Loss of taste*
stuffy_runny_nose	=>	*Stuffy and/or running nose*
loss_smell	=>	*Loss of smell*
dry_cough	=>	*Dry cough*
productive_cough	=>	*Productive cough*
short_breath	=>	*Shortness of breath*
body_pains	=>	*Muscle/body pains*
weakness	=>	*General weakness*
nausea	=>	*Nausea*
diarrhea	=>	*Diarrhea*
other	=>	*Other (specify)*
none	=>	*No symptoms*

Q: Specify other symptoms: (*)

Type: text

A: text input

Visible if

Q:	A:
:input[name="if_yes_which_symptoms_multiple_options_possib[other]"]	- checked => 1

Q: When did you experience these symptoms? (many answers possible) (*)

Type: choice_multiple

A: multiple answers possible:

two_weeks	=>	*During the last two weeks*
one_month	=>	*About 1 month ago*
2_3_months	=>	*Between 2-3 months ago*

more_3_months	=>	*More than 3 months ago*

Visible if

Q:	A:
:input[name="if_yes_which_symptoms_multiple_options_possib[none]"]	- unchecked => 1

Q: If you experienced any of these symptoms, were you or are you hospitalized for this illness? (*)

Type: choice

A: one of the following:

yes	=>	*Yes*
no	=>	*No*

Visible if

Q:	A:
:input[name="if_yes_which_symptoms_multiple_options_possib[none]"]	- unchecked => 1

Q: Have you been tested for COVID-19? (*)

Type: choice

A: one of the following:

once	=>	*Yes, once*
more_once	=>	*Yes, more than once*
no	=>	*No*

Q: When were you tested for COVID-19? (many answers possible) (*)

Type: choice_multiple

A: multiple answers possible:

two_weeks	=>	*During the last two weeks*
one_month	=>	*About 1 month ago*
2_3_months	=>	*Between 2-3 months ago*
more_3_months	=>	*More than 3 months ago*

Visible if

Q:	A:
Have you been tested for COVID-19?	- !value => no

Q: What initially motivated your COVID-19 testing? (*)

Type: choice

A: one of the following:

symptoms	=>	*I had symptoms of the disease*

contact	=>	*I was in contact with people who had the disease*
all_workers	=>	*All healthcare workers had to be tested*
worried	=>	*I had no symptoms or contacts, but was very worried and absolutely wanted to know my status*
other	=>	*Other*

Visible if

Q:	A:
Have you been tested for COVID-19?	- value => yes

Q: What was the result of your COVID-19 testing? (*)

Type: choice_scale

A: First testSecond testOther test(s)

0	=>	*Negative*
1	=>	*Positive*
NK	=>	*I don't know yet*
NA	=>	*Not applicable*

Visible if

Q:	A:
Have you been tested for COVID-19?	- !value => no

Q: If any of your COVID-19 tests was positive, what was done about it?

Type: choice

A: one of the following:

stop_work_home	=>	*I stopped working and stayed home*
stop_work_hosp	=>	*I stopped working and was hospitalized*
work_covid	=>	*I continued working, but only with COVID-19 patients*
work_usual	=>	*I continued working in my usual service with extra precautions*
nothing	=>	*Nothing was done*

Visible if

Q:	A:
0	- :input[name="what_was_the_result_of_your_covid_19_testing_[first]"] =>
1	
2	- :input[name="what_was_the_result_of_your_covid_19_testing_[second]"] =>
3	
4	- :input[name="what_was_the_result_of_your_covid_19_testing_[other]"] =>

Q: Did you get infected with COVID-19 and recovered from the disease? (*)

Type: choice

A: one of the following:

yes	=>	*Yes, I recovered and tested negative during follow-up*
still_recovering	=>	*I am still in the recovery phase*
no	=>	*No, I was never infected*
NA	=>	*Not applicable*

Q: In case you recovered from COVID-19, have you experienced any of the following symptoms since your recovery? (multiple options possible) (*)

Type: choice_multiple

A: multiple answers possible:

fever	=>	*Fever*
headache	=>	*Headache*
sore throat	=>	*Sore throat*
loss_taste	=>	*Loss of taste*
loss_smell	=>	*Loss of smell*
cough	=>	*Cough*
diff_breathing	=>	*Difficulties breathing*
body_pains	=>	*Muscle/body pains*
weakness	=>	*General weakness or fatigue*
insomnia	=>	*Difficulties to sleep*
tingling	=>	*Tingling sensations on your arms or legs*
memory_loss	=>	*Memory loss / difficulty remebering things*
other	=>	*Other symptoms*
none	=>	*No symptoms*

Visible if

Q:	A:
Did you get infected with COVID-19 and recovered from the disease?	- value => yes

Psychosocial well-being of healthcare workers

Part 1 (HADS-A): Tick the box beside the reply that is closest to how you have been feeling in the past week. Don’t take too long over your replies; your immediate answer is the best.

Q: I feel tense or wound up (*)

Type: choice

A: one of the following:

0	=>	*Not at all*
1	=>	*From time to time, occasionally*
2	=>	*Often*
3	=>	*Most of the time*

Q: I get a sort of frightened feeling as if something awful is about to happen(*)

Type: choice

A: one of the following:

3	=>	*Very definitely and quite badly*
2	=>	*Yes, but not too badly*
1	=>	*A little, but it does not worry me*
0	=>	*Not at all*

Q: Worrying thoughts go through my mind (*)

Type: choice

A: one of the following:

3	=>	*Most of the time*
2	=>	*Often*
1	=>	*From time to time, but not too often*
0	=>	*Only occasionally*

Q: I can sit at ease and feel relaxed (*)

Type: choice

A: one of the following:

0	=>	*Definitely*
1	=>	*Usually*
2	=>	*Not often*
3	=>	*Not at all*

Q: I get a sort of frightened feeling like knots in the stomach(*)

Type: choice

A: one of the following:

0	=>	*Not at all*
1	=>	*Occasionally*
2	=>	*Quite often*
3	=>	*Very often*

Q: I feel restless as if I have to be on the move(*)

Type: choice

A: one of the following:

3	=>	*Very much indeed*
2	=>	*Quite often*
1	=>	*Occasionally*
0	=>	*Not at all*

Q: I get sudden feelings of panic (*)

Type: choice

A: one of the following:

3	=>	*Very often indeed*
2	=>	*Quite often*
1	=>	*Occasionally*
0	=>	*Not at all*

Part 2 (HADS-D): Tick the box beside the reply that is closest to how you have been feeling in the past week. Don't take too long over your replies; your immediate answer is the best.

Q: I still enjoy the things I used to enjoy (*)

Type: choice

A: one of the following:

0	=>	*Definitely as much*
1	=>	*Not quite as much*
2	=>	*Only a little*
3	=>	*Hardly at all*

Q: I can laugh and see the funny side of things (*)

Type: choice

A: one of the following:

0	=>	*As much as I always could*
1	=>	*Not quite so much now*
2	=>	*Definitely not so much now*
3	=>	*Not at all*

Q: I feel cheerful (*)

Type: choice

A: one of the following:

3	=>	*Not at all*
2	=>	*Not often*
1	=>	*Sometimes*
0	=>	*Most of the time*

Q: I feel as if I am slowed down (*)

Type: choice

A: one of the following:

3	=>	*Nearly all the time*
2	=>	*Very often*
1	=>	*Sometimes*
0	=>	*Not at all*

Q: I have lost interest in my appearance (*)

Type: choice

A: one of the following:

3	=>	*Definitely*
2	=>	*I don't take as much care as I should*
1	=>	*I may not take quite as much care*
0	=>	*I take just as much care as ever*

Q: I look forward with enjoyment to things (*)

Type: choice

A: one of the following:

0	=>	*As much as I ever did*
1	=>	*Rather less than I used to*
2	=>	*Definitely less than I used to*
3	=>	*Hardly at all*

Q: I can enjoy a good book or radio or TV program (*)

Type: choice

A: one of the following:

0	=>	*Often*
1	=>	*Sometimes*
2	=>	*Not often*
3	=>	*Very seldom*

Consent

Q: I fully understand what this study is about, and I consent to participate. All the information I provide can be used by researchers to better understand the coronavirus epidemic and its impact on healthcare workers. (*)

Type: checkbox

A: checkbox

Q: (OPTIONAL) I consent to be re-contacted by the researchers for follow-up questions on this topic, and I register my email address for this purpose. My email address will only be used to anonymously re-contact me and to link follow-up surveys to this survey; it will not be accessed or seen by the research team.

Type: checkbox

A: checkbox

Q: Please type your email address here:

Type:
text

A: text input

Visible if

Q:	A:
(OPTIONAL) I consent to be re-contacted by the researchers for follow-up questions on this topic, and I register my email address for this purpose. My email address will only be used to anonymously re-contact me and to link follow-up surveys to this survey; it will not be accessed or seen by the research team.	- checked => 1
