# Supplementary material for: Impact of COVID-19 on Healthcare Workers in Brazil between August and November 2020: A Cross-Sectional Survey
Source: Int J Environ Res Public Health. 2021 Jun 17;18(12):6511. doi: 10.3390/ijerph18126511 (PMC8296453; doi:10.3390/ijerph18126511)
Supplement: Supplementary file 1 [file ijerph-18-06511-s001.zip › ijerph-1258440-supplementary/ijerph-1258440-tableS3.pdf]

COVID-19 survey for HCW [Round 2]

General questions

BACKGROUND

During the COVID-19 pandemic, health systems in many countries were overwhelmed, subjecting healthcare workers to greater workloads. In our first survey to study the impact of the pandemic on Brazilian HCW, we found that a high number of participants reported to have experienced a COVID-19 infection despite good adherence to COVID-19 preventive measures. Moreover, we noticed high rates of anxiety and depression. To better understand these findings we now invite you to participate in this second survey which also includes questions about vaccination against COVID-19.

Q: E-CONSENTING: I agree to participate in the study. (*)

Type: checkbox

A: checkbox

Q: Please confirm that you are a healthcare personnel or someone actively working in the healthcare sector(*)

Type: choice

A: one of the following:

yes	=>	*Yes, I confirm that I work in healthcare*
no	=>	*No, I am not involved in the health sector*

Q: Age (in years) (*)

Type: number

A: number (min: 1 / max: 110 / step: 1)

Q: Gender (*)

Type: choice

A: one of the following:

male	=>	*Male*
female	=>	*Female*

Q: Marital status (*)

Type: choice

A: one of the following:

single	=>	*Single*
cohabit	=>	*Cohabitation*
legally_married	=>	*Legally married*
divorced	=>	*Divorced*
widow_widower	=>	*Widow/widower*

not_married_not_cohab_but_in_relationship	=>	*I am not married, nor in cohabitation, but I am in a relationship*

Q: Which country do you currently live in? (*)

Type: choice

A: one of the following:

Thailand	=>	*Thailand*
Afghanistan	=>	*Afghanistan*
Albania	=>	*Albania*
Algeria	=>	*Algeria*
American Samoa	=>	*American Samoa*
Andorra	=>	*Andorra*
Angola	=>	*Angola*
Anguilla	=>	*Anguilla*
Antarctica	=>	*Antarctica*
Antigua & Barbuda	=>	*Antigua & Barbuda*
Argentina	=>	*Argentina*
Armenia	=>	*Armenia*
Aruba	=>	*Aruba*
Ascension Island	=>	*Ascension Island*
Australia	=>	*Australia*
Austria	=>	*Austria*
Azerbaijan	=>	*Azerbaijan*
Bahamas	=>	*Bahamas*
Bahrain	=>	*Bahrain*
Bangladesh	=>	*Bangladesh*
Barbados	=>	*Barbados*
Belarus	=>	*Belarus*
Belgium	=>	*Belgium*
Belize	=>	*Belize*
Benin	=>	*Benin*
Bermuda	=>	*Bermuda*
Bhutan	=>	*Bhutan*
Bolivia	=>	*Bolivia*
Bosnia & Herzegovina	=>	*Bosnia & Herzegovina*
Botswana	=>	*Botswana*
Bouvet Island	=>	*Bouvet Island*
Brazil	=>	*Brazil*
British Indian Ocean Territory	=>	*British Indian Ocean Territory*
British Virgin Islands	=>	*British Virgin Islands*
Brunei	=>	*Brunei*
Bulgaria	=>	*Bulgaria*
Burkina Faso	=>	*Burkina Faso*
Burundi	=>	*Burundi*
Cambodia	=>	*Cambodia*

Cameroon	=>	*Cameroon*
Canada	=>	*Canada*
Canary Islands	=>	*Canary Islands*
Cape Verde	=>	*Cape Verde*
Caribbean Netherlands	=>	*Caribbean Netherlands*
Cayman Islands	=>	*Cayman Islands*
Central African Republic	=>	*Central African Republic*
Ceuta & Melilla	=>	*Ceuta & Melilla*
Chad	=>	*Chad*
Chile	=>	*Chile*
China	=>	*China*
Christmas Island	=>	*Christmas Island*
Clipperton Island	=>	*Clipperton Island*
Cocos (Keeling) Islands	=>	*Cocos (Keeling) Islands*
Colombia	=>	*Colombia*
Comoros	=>	*Comoros*
Congo - Brazzaville	=>	*Congo - Brazzaville*
Congo - Kinshasa	=>	*Congo - Kinshasa*
Cook Islands	=>	*Cook Islands*
Costa Rica	=>	*Costa Rica*
Croatia	=>	*Croatia*
Cuba	=>	*Cuba*
Curaçao	=>	*Curaçao*
Cyprus	=>	*Cyprus*
Czechia	=>	*Czechia*
Côte d’Ivoire	=>	*Côte d’Ivoire*
Denmark	=>	*Denmark*
Diego Garcia	=>	*Diego Garcia*
Djibouti	=>	*Djibouti*
Dominica	=>	*Dominica*
Dominican Republic	=>	*Dominican Republic*
Ecuador	=>	*Ecuador*
Egypt	=>	*Egypt*
El Salvador	=>	*El Salvador*
Equatorial Guinea	=>	*Equatorial Guinea*
Eritrea	=>	*Eritrea*
Estonia	=>	*Estonia*
Eswatini	=>	*Eswatini*
Ethiopia	=>	*Ethiopia*
Falkland Islands	=>	*Falkland Islands*
Faroe Islands	=>	*Faroe Islands*
Fiji	=>	*Fiji*
Finland	=>	*Finland*
France	=>	*France*
French Guiana	=>	*French Guiana*
French Polynesia	=>	*French Polynesia*

French Southern Territories	=>	*French Southern Territories*
Gabon	=>	*Gabon*
Gambia	=>	*Gambia*
Georgia	=>	*Georgia*
Germany	=>	*Germany*
Ghana	=>	*Ghana*
Gibraltar	=>	*Gibraltar*
Greece	=>	*Greece*
Greenland	=>	*Greenland*
Grenada	=>	*Grenada*
Guadeloupe	=>	*Guadeloupe*
Guam	=>	*Guam*
Guatemala	=>	*Guatemala*
Guernsey	=>	*Guernsey*
Guinea	=>	*Guinea*
Guinea-Bissau	=>	*Guinea-Bissau*
Guyana	=>	*Guyana*
Haiti	=>	*Haiti*
Heard & McDonald Islands	=>	*Heard & McDonald Islands*
Honduras	=>	*Honduras*
Hong Kong SAR China	=>	*Hong Kong SAR China*
Hungary	=>	*Hungary*
Iceland	=>	*Iceland*
India	=>	*India*
Indonesia	=>	*Indonesia*
Iran	=>	*Iran*
Iraq	=>	*Iraq*
Ireland	=>	*Ireland*
Isle of Man	=>	*Isle of Man*
Israel	=>	*Israel*
Italy	=>	*Italy*
Jamaica	=>	*Jamaica*
Japan	=>	*Japan*
Jersey	=>	*Jersey*
Jordan	=>	*Jordan*
Kazakhstan	=>	*Kazakhstan*
Kenya	=>	*Kenya*
Kiribati	=>	*Kiribati*
Kosovo	=>	*Kosovo*
Kuwait	=>	*Kuwait*
Kyrgyzstan	=>	*Kyrgyzstan*
Laos	=>	*Laos*
Latvia	=>	*Latvia*
Lebanon	=>	*Lebanon*
Lesotho	=>	*Lesotho*
Liberia	=>	*Liberia*

Libya	=>	*Libya*
Liechtenstein	=>	*Liechtenstein*
Lithuania	=>	*Lithuania*
Luxembourg	=>	*Luxembourg*
Macao SAR China	=>	*Macao SAR China*
Madagascar	=>	*Madagascar*
Malawi	=>	*Malawi*
Malaysia	=>	*Malaysia*
Maldives	=>	*Maldives*
Mali	=>	*Mali*
Malta	=>	*Malta*
Marshall Islands	=>	*Marshall Islands*
Martinique	=>	*Martinique*
Mauritania	=>	*Mauritania*
Mauritius	=>	*Mauritius*
Mayotte	=>	*Mayotte*
Mexico	=>	*Mexico*
Micronesia	=>	*Micronesia*
Moldova	=>	*Moldova*
Monaco	=>	*Monaco*
Mongolia	=>	*Mongolia*
Montenegro	=>	*Montenegro*
Montserrat	=>	*Montserrat*
Morocco	=>	*Morocco*
Mozambique	=>	*Mozambique*
Myanmar (Burma)	=>	*Myanmar (Burma)*
Namibia	=>	*Namibia*
Nauru	=>	*Nauru*
Nepal	=>	*Nepal*
Netherlands	=>	*Netherlands*
Netherlands Antilles	=>	*Netherlands Antilles*
New Caledonia	=>	*New Caledonia*
New Zealand	=>	*New Zealand*
Nicaragua	=>	*Nicaragua*
Niger	=>	*Niger*
Nigeria	=>	*Nigeria*
Niue	=>	*Niue*
Norfolk Island	=>	*Norfolk Island*
Northern Mariana Islands	=>	*Northern Mariana Islands*
North Korea	=>	*North Korea*
North Macedonia	=>	*North Macedonia*
Norway	=>	*Norway*
Oman	=>	*Oman*
Outlying Oceania	=>	*Outlying Oceania*
Pakistan	=>	*Pakistan*
Palau	=>	*Palau*

Palestinian Territories	=>	*Palestinian Territories*
Panama	=>	*Panama*
Papua New Guinea	=>	*Papua New Guinea*
Paraguay	=>	*Paraguay*
Peru	=>	*Peru*
Philippines	=>	*Philippines*
Pitcairn Islands	=>	*Pitcairn Islands*
Poland	=>	*Poland*
Portugal	=>	*Portugal*
Puerto Rico	=>	*Puerto Rico*
Qatar	=>	*Qatar*
Romania	=>	*Romania*
Russia	=>	*Russia*
Rwanda	=>	*Rwanda*
Réunion	=>	*Réunion*
Samoa	=>	*Samoa*
San Marino	=>	*San Marino*
Saudi Arabia	=>	*Saudi Arabia*
Senegal	=>	*Senegal*
Serbia	=>	*Serbia*
Seychelles	=>	*Seychelles*
Sierra Leone	=>	*Sierra Leone*
Singapore	=>	*Singapore*
Sint Maarten	=>	*Sint Maarten*
Slovakia	=>	*Slovakia*
Slovenia	=>	*Slovenia*
Solomon Islands	=>	*Solomon Islands*
Somalia	=>	*Somalia*
South Africa	=>	*South Africa*
South Georgia & South Sandwich Islands	=>	*South Georgia & South Sandwich Islands*
South Korea	=>	*South Korea*
South Sudan	=>	*South Sudan*
Spain	=>	*Spain*
Sri Lanka	=>	*Sri Lanka*
St. Barthélemy	=>	*St. Barthélemy*
St. Helena	=>	*St. Helena*
St. Kitts & Nevis	=>	*St. Kitts & Nevis*
St. Lucia	=>	*St. Lucia*
St. Martin	=>	*St. Martin*
St. Pierre & Miquelon	=>	*St. Pierre & Miquelon*
St. Vincent & Grenadines	=>	*St. Vincent & Grenadines*
Sudan	=>	*Sudan*
Suriname	=>	*Suriname*
Svalbard & Jan Mayen	=>	*Svalbard & Jan Mayen*
Sweden	=>	*Sweden*

Switzerland	=>	*Switzerland*
Syria	=>	*Syria*
São Tomé & Príncipe	=>	*São Tomé & Príncipe*
Taiwan	=>	*Taiwan*
Tajikistan	=>	*Tajikistan*
Tanzania	=>	*Tanzania*
Timor-Leste	=>	*Timor-Leste*
Togo	=>	*Togo*
Tokelau	=>	*Tokelau*
Tonga	=>	*Tonga*
Trinidad & Tobago	=>	*Trinidad & Tobago*
Tristan da Cunha	=>	*Tristan da Cunha*
Tunisia	=>	*Tunisia*
Turkey	=>	*Turkey*
Turkmenistan	=>	*Turkmenistan*
Turks & Caicos Islands	=>	*Turks & Caicos Islands*
Tuvalu	=>	*Tuvalu*
U.S. Outlying Islands	=>	*U.S. Outlying Islands*
U.S. Virgin Islands	=>	*U.S. Virgin Islands*
Uganda	=>	*Uganda*
Ukraine	=>	*Ukraine*
United Arab Emirates	=>	*United Arab Emirates*
United Kingdom	=>	*United Kingdom*
United States	=>	*United States*
Uruguay	=>	*Uruguay*
Uzbekistan	=>	*Uzbekistan*
Vanuatu	=>	*Vanuatu*
Vatican City	=>	*Vatican City*
Venezuela	=>	*Venezuela*
Vietnam	=>	*Vietnam*
Wallis & Futuna	=>	*Wallis & Futuna*
Western Sahara	=>	*Western Sahara*
Yemen	=>	*Yemen*
Zambia	=>	*Zambia*
Zimbabwe	=>	*Zimbabwe*
Åland Islands	=>	*Åland Islands*

Q: In which state of Brazil do you live? (*)

Type: choice

A: one of the following:

Acre	=>	*Acre*
Alagoas	=>	*Alagoas*
Amapá	=>	*Amapá*
Amazonas	=>	*Amazonas*
Bahia	=>	*Bahia*

Ceará	=>	*Ceará*
Distrito Federal	=>	*Distrito Federal*
Espírito Santo	=>	*Espírito Santo*
Goiás	=>	*Goiás*
Maranhão	=>	*Maranhão*
Mato Grosso	=>	*Mato Grosso*
Mato Grosso do Sul	=>	*Mato Grosso do Sul*
Minas Gerais	=>	*Minas Gerais*
Pará	=>	*Pará*
Paraíba	=>	*Paraíba*
Paraná	=>	*Paraná*
Pernambuco	=>	*Pernambuco*
Piauí	=>	*Piauí*
Rio de Janeiro	=>	*Rio de Janeiro*
Rio Grande do Norte	=>	*Rio Grande do Norte*
Rio Grande do Sul	=>	*Rio Grande do Sul*
Rondônia	=>	*Rondônia*
Roraima	=>	*Roraima*
Santa Catarina	=>	*Santa Catarina*
São Paulo	=>	*São Paulo*
Sergipe	=>	*Sergipe*
Tocantins	=>	*Tocantins*

Visible if

Q:	A:
0	- :input[name="which_country_do_you_currently_live_in_"] =>
1	
2	- :input[name="which_country_do_you_currently_live_in_"] =>

Q: Which city/village do you live in? (*)

Type: text

A: text input

Work-related questions

Q: Which organisation do you work for? (*)

Type: choice

A: one of the following:

government	=>	*Public (government)*
private	=>	*Private (individual or NGO)*
other	=>	*Other (Specify)*

Q: Please specify other type of organisation: (*)

Type: text

A: text input

Visible if

Q:	A:
Which organisation do you work for?	- value => other

Q: Which hospital or other health structure are you working in? (*)

Type: text

A: text input

Q: What is your profession? (*)

Type: choice

A: one of the following:

chw	=>	*Community Health Worker*
nurse	=>	*Nurse*
clinical_officer	=>	*Clinical Officer*
laboratory	=>	*Laboratory staff*
pharmacy	=>	*Pharmacy staff*
generalist	=>	*Medical doctor: general practitioner*
specialist	=>	*Medical doctor: specialist*
doctor_other	=>	*Medical doctor: other*
administrative	=>	*Administrative person working in a hospital or clinic*
dentist	=>	*Dentist*
psychologist	=>	*Psychologist*
support_staff	=>	*Support staff working in a hospital or clinic (driver, cleaner, maintenance, etc)*
finance	=>	*Finance department*
physiotherapist	=>	*Physiotherapist*
biomedical	=>	*Biomedical*
social_worker	=>	*Social worker*
radio_tech	=>	*Radiology technician*
nurse_tech	=>	*Nursing technician*
other	=>	*Other (please specify)*

Q: Please specify other profession:

Type: text

A: text input

Visible if

Q:	A:
What is your profession?	- value => other

Q: Are you directly involved in patient care, with physical contact? (*)

Type: choice

A: one of the following:

yes_touch	=>	*Yes, and I touch patients*
yes_notouch	=>	*Yes, but I do not touch patient (proxy for no close contact)*
no_earlier	=>	*No, but I had physical contact earlier during the COVID-19 pandemic*
no_never	=>	*No, and I never had physical contact during the COVID-19 pandemic*

Q: If yes, in which service do you work? (multiple answers possible) (*)

Type: choice_multiple

A: multiple answers possible:

hospitalisation	=>	*Patient hospitalisation ward/internal medicine ward*
other_ward	=>	*In another ward (surgery, pediatrics, obstetrics/gynaecology)*
icu	=>	*Intensive Care Unit (ICU)*
emergency	=>	*The emergency department*
outpatient	=>	*Outpatient clinic / department*
other	=>	*Other (specify, even if it is outside the hospital)*

Visible if

Q:	A:
0	- :input[name="are_you_involved_in_health_care_"] =>
1	
2	- :input[name="are_you_involved_in_health_care_"] =>

Q: Please specify other service where you work:

Type: text

A: text input

Visible if

Q:	A:
:input[name="if_yes_in_which_service_do_you_work_multiple_answers_possible_[other]"]	- checked => 1

Q: When do you wear a face mask? (*)

Type: choice

A: one of the following:

only_hospital	=>	*Only in the hospital / clinic*
hosp_out_occasionally	=>	*In the hospital and occasionally when I went outside (less than half of the times)*
hosp_out_sometimes	=>	*In the hospital and sometimes when I went outside (more than half of the times)*
hosp_out_always	=>	*In the hospital and anytime I went outside*
never	=>	*Never*

Hospital work during the pandemic

PLEASE ANSWER THE FOLLOWING QUESTIONS BASED ON THE PAST THREE WEEKS.

Q: How many days did you go to work each week? (*)

Type: number

A: number (min: n/a / max: 7 / step: 1)

Q: How many hours (on average) did you spend at work each day? (*)

Type: number

A: number (min: n/a / max: 24 / step: n/a)

Q: Have your tasks in the hospital / clinic changed? (e.g. shifted to another ward or department, etc) (*)

Type: choice

A: one of the following:

yes	=>	*Yes*
no	=>	*No*
na	=>	*Not applicable*

Q: If yes, what was the change? (multiple answers possible) (*)

Type: choice_multiple

A: multiple answers possible:

covid_ward	=>	*I am now assisting in a ward with COVID patients*
icu	=>	*I am now assisting at the intensive care unit*
emergency	=>	*I am now assisting at the emergency department*
other	=>	*Other (specify)*

Visible if

Q:	A:
Have your tasks in the hospital / clinic changed? (e.g. shifted to another ward or department, etc)	- value => yes

Q: Specify other changes at work:

Type: text

A: text input

Visible if

Q:	A:
:input[name="if_yes_what_was_the_change_[other]"]	- checked => 1

Q: Has your salary/income changed? (*)

Type: choice

A: one of the following:

yes	=>	*Yes*
no	=>	*No*
na	=>	*Not applicable*

Q: If yes, specify why the change in salary / income:

Type: text

A: text input

Visible if

Q:	A:
Has your salary/income changed?	- value => yes

Q: Which type of mask did you wear in the hospital / clinic while attending to patients? (*)

Type: choice

A: one of the following:

surgical	=>	*A surgical mask*
cloth	=>	*A cloth mask*
respirator	=>	*A respirator (N95 or FFP2/3 mask)*
respirator_cloth	=>	*Sometimes a respirator (N95 or FFP2/3 mask), other times a cloth mask*
surgical_cloth	=>	*Sometimes a surgical mask, other times a cloth mask*
none	=>	*No mask*

Q: How often did you change your mask while in your work environment (hospital / clinic)?(*)

Type: choice

A: one of the following:

never	=>	*Never*
once_week	=>	*1 time per week*
2_4_times_week	=>	*2-4 times per week*
5_6_times_week	=>	*5-6 times per week*

daily	=>	*Once a day*
multiple_daily	=>	*Multiple times a day*

Q: Which type of mask did you wear outside of your work environment? (*)

Type: choice

A: one of the following:

surgical	=>	*A surgical mask*
cloth	=>	*A cloth mask*
respirator	=>	*A respirator (N95 or FFP2/3 mask)*
respirator_cloth	=>	*Sometimes a respirator (N95 or FFP2/3 mask), other times a cloth mask*
surgical_cloth	=>	*Sometimes a surgical mask, other times a cloth mask*
none	=>	*No mask use outside the hospital / clinic*

Q: If you wear a face mask outside your work environment, how often did you change your mask?(*)

Type: choice

A: one of the following:

never	=>	*Never*
once_week	=>	*1 time per week*
2_4_times_week	=>	*2-4 times per week*
5_6_times_week	=>	*5-6 times per week*
daily	=>	*Once a day*
multiple_daily	=>	*Multiple times a day*

Q: If you use cloth masks, how do you wash them? (*)

Type: choice

A: one of the following:

manual_tap	=>	*Manually with tap water but without boiling*
manual_boiled	=>	*Manually with tap water, and I boil my mask in water*
manual_notap_noboil	=>	*Manually, without tap water and without boiling*
manual_notap_boil	=>	*Manually, without tap water and I boil my mask*
machine_low	=>	*In a washing machine at low temperature*
machine_high	=>	*In a washing machine at high temperature*
not_cloth	=>	*I do not use cloth masks*

Q: Does your work environment have a specific patient screening strategy for suspect and non-suspect COVID-19 patients? (*)

Type: choice

A: one of the following:

yes	=>	*Yes*

no	=>	*No*

Q: Have you been in close contact (<2 metres distance) with patients suspected to have COVID-19 in the hospital?(*)

Type: choice

A: one of the following:

yes	=>	*Yes*
no	=>	*No*
dont_know	=>	*I don't know*

Q: If yes, did you wash hands, or used hand sanitizer before and after you saw each patient?(*)

Type: choice

A: one of the following:

yes	=>	*Yes*
no_time	=>	*No: I wanted to, but there was no time to wash hands*
no_water_soap	=>	*No: I wanted to, but there was no water/soap/hand sanitizer*
no_forgot	=>	*No, I forgot to wash my hands*
no_not_necessary	=>	*No, I don't think it is necessary to do this all the time*

Visible if

Q:	A:
Have you been in close contact (<2 metres distance) with patients suspected to have COVID-19 in the hospital?	- value => yes

Q: Did you wear a protective apron? (*)

Type: choice

A: one of the following:

yes_daily	=>	*Yes, and I changed my apron every day*
yes_not_daily	=>	*Yes, but I could not change my apron every day*
no_aprons	=>	*No, there were no aprons available*
no_not_necessary	=>	*No, I don't think it is necessary to wear an apron*

Visible if

Q:	A:
Have you been in close contact (<2 metres distance) with patients suspected to have COVID-19 in the hospital?	- value => yes

Q: Did you wear a face mask? (*)

Type: choice

A: one of the following:

yes_daily	=>	*Yes, and I changed my mask every day*
yes_not_daily	=>	*Yes, but I could not change my mask every day*
no_masks	=>	*No, there were no masks available*
no_not_necessary	=>	*No, I didn't think it was necessary to wear a mask*

Visible if

Q:	A:
Have you been in close contact (<2 metres distance) with patients suspected to have COVID-19 in the hospital?	- value => yes

Q: Did you wear protective goggles or face shield when attending to patients? (*)

Type: choice

A: one of the following:

yes	=>	*Yes*
no_glasses	=>	*No, but I wear normal eyeglasses*
no	=>	*No, my eyes are unprotected*
not_applicable	=>	*Not applicable (I do not attend to patients)*

Health-related questions

Q: Since your last participation in this online survey, have you experienced any of the following flu-like symptoms? (multiple options possible) (*)

Type: choice_multiple

A: multiple answers possible:

fever	=>	*Fever*
headache	=>	*Headache*
sore throat	=>	*Sore throat*
dry_cough	=>	*Dry cough*
productive_cough	=>	*Productive cough*
short_breath	=>	*Shortness of breath*
body_pains	=>	*Muscle/body pains*
weakness	=>	*General weakness*
nausea	=>	*Nausea*
diarrhea	=>	*Diarrhea*
loss_taste	=>	*Loss of taste*
stuffy_blocked_nose	=>	*Stuffy and/or blocked nose*
runny_nose	=>	*Running nose*
loss_smell	=>	*Loss of smell / Abnormal smell*
none	=>	*No symptoms*

Q: If you developed flu-like symptoms, when did these symptoms appear? (*)

Type: date

A: date input

Visible if

Q:	A:
:input[name="if_yes_which_symptoms_multiple_options_possib[none]"]	- unchecked => 1

Q: How many days did the symptoms last? (*)

Type: number

A: number (min: 1 / max: n/a / step: 1)

Visible if

Q:	A:
:input[name="if_yes_which_symptoms_multiple_options_possib[none]"]	- unchecked => 1

Q: If you experienced any of these symptoms, were you or are you hospitalized for this illness? (*)

Type: choice

A: one of the following:

yes	=>	*Yes*
no	=>	*No*

Visible if

Q:	A:
:input[name="if_yes_which_symptoms_multiple_options_possib[none]"]	- unchecked => 1

Q: If abnormal smell / loss of smell, how would you describe it?(*)

Type: choice

A: one of the following:

distorted	=>	*My sense of smell is distorted (things smell peculiar)*
phantom	=>	*Experience a smell when nothing is there (phantom smell)*
hypersensitive	=>	*My sense of smell is heightened (hypersensitive)*
partial_loss	=>	*My sense of smell is diminished (partial loss)*
complete_loss	=>	*My sense of smell is absent (complete loss)*

Visible if

Q:	A:
:input[name="if_yes_which_symptoms_multiple_options_possib[loss_smell]"]	- checked => 1

Q: Did you experience an episode of fatigue without fever but with loss of smell?(*)

Type: choice

A: one of the following:

yes	=>	*Yes*
no	=>	*No*

Q: Have you been tested for COVID-19? (*)

Type: choice

A: one of the following:

once	=>	*Yes, once*
more_once	=>	*Yes, more than once*
no	=>	*No*

Q: If yes, when was the test done? (many answers possible) (*)

Type: choice_multiple

A: multiple answers possible:

two_weeks	=>	*During the last two weeks*
one_month	=>	*About 1 month ago*
2_3_months	=>	*Between 2-3 months ago*
more_3_months	=>	*More than 3 months ago*

Visible if

Q:	A:
Have you been tested for COVID-19?	- !value => no

Q: If yes, why was the test done? (*)

Type: choice

A: one of the following:

symptoms	=>	*Because of illness*
contact	=>	*Because of a contact with a person with confirmed COVID -19 infection*
all_workers	=>	*Because all healthcare workers were tested*
other	=>	*Other reason*

Visible if

Q:	A:
Have you been tested for COVID-19?	- value => yes

Q: What was the result of your COVID-19 test? (*)

Type: choice

A: one of the following:

positive	=>	*Positive*

negative	=>	*Negative*
do_not_know	=>	*Do not know yet*

Visible if

Q:	A:
Have you been tested for COVID-19?	- value => yes

Q: If your COVID-19 test was positive, what was done about it? (*)

Type: choice

A: one of the following:

stop_work	=>	*I stopped working*
work_covid	=>	*I continued working, but only with COVID-19 patients*
nothing	=>	*Nothing was done*

Visible if

Q:	A:
0	- :input[name="what_was_the_result_of_your_covid_19_testing_[first]"] =>
1	
2	- :input[name="what_was_the_result_of_your_covid_19_testing_[second]"] =>
3	
4	- :input[name="what_was_the_result_of_your_covid_19_testing_[other]"] =>

COVID-19 Vaccination

Q: In your opinion, can COVID-19 infection be prevented by a vaccine? (*)

Type: choice

A: one of the following:

yes	=>	*Yes*
no	=>	*No*
na	=>	*I don't know*

Q: To the best of your knowledge, is there currently an effective vaccine against COVID-19? (*)

Type: choice

A: one of the following:

yes	=>	*Yes*
no	=>	*No*
na	=>	*I don't know*

Q: Would you be willing to take the COVID-19 vaccine when it becomes available? (*)

Type: choice_scale

A: If it is at least 50% effectiveIf it is at least 75% effectiveIf it is at least 90% effectiveIf it is at least 95% effective

1	=>	*Yes*
0	=>	*No*
na	=>	*No opinion*

Q: What are some of the possible reasons why you would hesitate to take the COVID-19 vaccine? (many answers possible) (*)

Type: choice_multiple

A: multiple answers possible:

covid_unreal	=>	*I don't think COVID-19 exists*
not_effective	=>	*I think the vaccine is not effective*
harm	=>	*I think the vaccine is designed to harm us*
side_effects	=>	*I am scared of side-effects of the vaccine*
not_needed	=>	*My body is naturally strong, I don't need a vaccine to fight COVID-19*
immune	=>	*I already had COVID-19, so I think I am immune to the disease*
covid_finished	=>	*The COVID-19 pandemic is finished in my country, no need for a vaccine now*
none	=>	*None of the above*
other	=>	*Other reasons (please specify)*

Q: Please specify other reasons for hesitating to receive the COVID-19 vaccine: (*)

Type: text

A: text input

Visible if

Q:	A:
:input[name="what_are_some_of_the_possible_reasons_why_you_would_hesitate_to_[other]"]	- checked => 1

Q: If you are willing to be vaccinated, would you accept to be vaccinated with the Chinese vaccine?(*)

Type: choice

A: one of the following:

yes	=>	*Yes*
no	=>	*No*
na	=>	*I don't know*

Q: How important is it to you that by getting the COVID-19 vaccine, you would protect your own health?(*)

Type: choice_scale

A: On a scale of 1 to 5

1	=>	*1=Not at all important*

2	=>	*2=A little important*
3	=>	*3=Moderately important*
4	=>	*4=Very important*
5	=>	*5=Extremely important*

Q: How important is it to you that by getting the COVID-19 vaccine, you would protect the health of other people in your community? (*)

Type: choice_scale

A: On a scale of 1 to 5

1	=>	*1=Not at all important*
2	=>	*2=A little important*
3	=>	*3=Moderately important*
4	=>	*4=Very important*
5	=>	*5=Extremely important*

Psychosocial well-being of healthcare workers

Part 1 (HADS-A): Tick the box beside the reply that is closest to how you have been feeling in the past week. Don’t take too long over your replies; your immediate answer is the best.

Q: I feel tense or wound up (*)

Type: choice

A: one of the following:

0	=>	*Not at all*
1	=>	*From time to time, occasionally*
2	=>	*Often*
3	=>	*Most of the time*

Q: I get a sort of frightened feeling as if something awful is about to happen(*)

Type: choice

A: one of the following:

3	=>	*Very definitely and quite badly*
2	=>	*Yes, but not too badly*
1	=>	*A little, but it does not worry me*
0	=>	*Not at all*

Q: Worrying thoughts go through my mind (*)

Type: choice

A: one of the following:

3	=>	*Most of the time*
2	=>	*Often*
1	=>	*From time to time, but not too often*
0	=>	*Only occasionally*

Q: I can sit at ease and feel relaxed (*)

Type: choice

A: one of the following:

0	=>	*Definitely*
1	=>	*Usually*
2	=>	*Not often*
3	=>	*Not at all*

Q: I get a sort of frightened feeling like knots in the stomach(*)

Type: choice

A: one of the following:

0	=>	*Not at all*
1	=>	*Occasionally*
2	=>	*Quite often*
3	=>	*Very often*

Q: I feel restless as if I have to be on the move (*)

Type: choice

A: one of the following:

3	=>	*Very much indeed*
2	=>	*Quite often*
1	=>	*Occasionally*
0	=>	*Not at all*

Q: I get sudden feelings of panic (*)

Type: choice

A: one of the following:

3	=>	*Very often indeed*
2	=>	*Quite often*
1	=>	*Occasionally*
0	=>	*Not at all*

Part 2 (HADS-D): Tick the box beside the reply that is closest to how you have been feeling in the past week. Don’t take too long over your replies; your immediate answer is the best.

Q: I still enjoy the things I used to enjoy (*)

Type: choice

A: one of the following:

0	=>	*Definitely as much*
1	=>	*Not quite as much*
2	=>	*Only a little*
3	=>	*Hardly at all*

Q: I can laugh and see the funny side of things (*)

Type: choice

A: one of the following:

0	=>	*As much as I always could*
1	=>	*Not quite so much now*
2	=>	*Definitely not so much now*
3	=>	*Not at all*

Q: I feel cheerful (*)

Type: choice

A: one of the following:

3	=>	*Not at all*
2	=>	*Not often*
1	=>	*Sometimes*
0	=>	*Most of the time*

Q: I feel as if I am slowed down (*)

Type: choice

A: one of the following:

3	=>	*Nearly all the time*
2	=>	*Very often*
1	=>	*Sometimes*
0	=>	*Not at all*

Q: I have lost interest in my appearance (*)

Type: choice

A: one of the following:

3	=>	*Definitely*
2	=>	*I don't take as much care as I should*

1	=>	*I may not take quite as much care*
0	=>	*I take just as much care as ever*

Q: I look forward with enjoyment to things (*)

Type: choice

A: one of the following:

0	=>	*As much as I ever did*
1	=>	*Rather less than I used to*
2	=>	*Definitely less than I used to*
3	=>	*Hardly at all*

Q: I can enjoy a good book or radio or TV program (*)

Type: choice

A: one of the following:

0	=>	*Often*
1	=>	*Sometimes*
2	=>	*Not often*
3	=>	*Very seldom*

Q: I fully understand what this study is about, and I consent to participate. All the information I provide can be used by researchers to better understand the coronavirus epidemic and its impact on healthcare workers. (*)

Type: checkbox

A: checkbox

Q: (OPTIONAL) I consent to be re-contacted by the researchers for follow-up questions on this topic, and I register my email address for this purpose. My email address will only be used to anonymously re-contact me and to link follow-up surveys to this survey; it will not be accessed or seen by the research team.

Type: checkbox

A: checkbox

Q: Please type your email address here:

Type: text

A: text input

Visible if

Q:	A:
(OPTIONAL) I consent to be re-contacted by the researchers for follow-up questions on this topic, and I register my email address for this purpose. My email address will only be used to anonymously re-contact me and to link follow-up surveys to this survey; it will not be accessed or seen by the research team.	- checked => 1
