# Supplementary material for: Impact of COVID-19 on Healthcare Workers in Brazil between August and November 2020: A Cross-Sectional Survey
Source: Int J Environ Res Public Health. 2021 Jun 17;18(12):6511. doi: 10.3390/ijerph18126511 (PMC8296453; doi:10.3390/ijerph18126511)
Supplement: Supplementary file 1 [file ijerph-18-06511-s001.zip › Table S1.pdf]

Supplementary table

Supplementary Table S1. Multivariate logistic regression model for factors associated with a COVID-19 positive test

**Covariates**	**Negative test(n=246)**	**Positive test(n=49)**	**Crude OR (95% CI)**	**Adjusted OR (95% CI)**	**P-value**
Number of flu-like symptoms: Mean ± SD	0 (0 – 2)	0 (0-5)	1.16 (1.05 - 1.28)	1.17 (1.06 – 1.29)	**0.002**
Hospital Restructured Due to COVID-19					
No	75 (30.5%)	13 (26.5%)	Ref	Ref	0.380
Yes	171 (69.5 %)	36 (73.5 %)	1.21 (0.61 - 2.42)	1.37 (0.67 – 2.79)	

OR: Odds ratio; CI: Confidence interval; Ref: Reference category. IQR: Interquartile range
